# Collectanea Medica: Consisting of Anecdotes, Facts, Extracts, Illustrations, &c.

**Published:** 1824-03

**Authors:** 


### COLLECTANEA MEDICA:

CONSISTING OF

### ANECDOTES, FACTS, EXTRACTS, ILLUSTRATIONS, &c.

*Relating to the History or the Art of Medicine, and the Collateral Sciences.*

Floriferis, ut apes, in saltibus omnia libant,

Omnia nos, itidem, depascimur aurea dicta.

---

ART. I.—*Extracts from "Medical Jurisprudence."* By J. A. PARIS, M.D. &c. and J. S. M. FONBLANQUE, Esq.

### *Rape.*

RAPE is the unlawful and carnal knowledge of a woman by force and against her will; a ravishment of the body and violent deflowering her, which is felony by the common and statute law. Formerly it was the law, (especially in cases of appeals of rape,) in order to prevent malicious accusations, that the woman should immediately after, "*dum recens fuerit maleficium,*" go to the next town, and there make discovery to some credible persons of the injury she had received; and afterwards acquaint the high constable of the hundred, the coroners, and the sheriff, with the outrage. Afterwards, by statute *Westm.* 1. 3. Ed. I. c. 13, the time of limitation was extended to forty days. At present there is no time of limitation fixed; for it is punished at the suit of the king, and the maxim of the law takes place, that *nullum tempus occurrit regi.* The appeal of rape (for there were formerly several appeals beside that of murder,) has been long obsolete; and is now abolished by the statute 59 Geo. III. c. 46. But, though there is no time limited, a jury will seldom give credit to a stale complaint. In Scotland, it is said the limit was twenty-four hours; and, in a medical point of view, it is yet more necessary that examination should be immediate: many collateral proofs might be observed on an early inquiry, all signs of which would be obliterated in a few hours.* This remark

---

* The injuries thus occasioned consist in rupture of the hymen; swelling, contusion, inflammation, or laceration of the parts; discharge of blood; and, in persons of extreme youth, the laceration of the perineum is said to have sometimes occurred; and, as rape cannot be completed without considerable violence, we should also expect to find marks of force in other parts of the body, such as bruises about the arms and thighs: but, in appreciating the value of such indications, let the practitioner remember that the greater part of them may occur where the connexion has taken place with the consent of the female, or they may even be the effect of disease. Dr. Percival relates a case where the inflammation of the pudenda and symptoms of defloration occurred in a child four years old, which occasioned her death: there were strong reasons for suspecting that she had been injured by a boy of fourteen years of age, and he was accordingly taken into custody; but the case received elucidation from several others of a similar nature having been shortly afterwards received into the same hospital, and of whose nature no doubt could be entertained. When rape has been committed, gonorrhœa or lues venera are sometimes communicated, especially in cases of applies as well to the supposed criminal as to the sufferer; both should, in all possible cases, be subjected to immediate surgical examination: the case related by Sir Matthew Hale *(P.C.)* furnishes an instance where an innocent man might have been saved from a malicious prosecution to the hazard of his life, by this precaution. Foderé, in his work on Medical Jurisprudence, (vol. iv. p. 363,) mentions two cases from Zacchias, where the falsehood of an accusation was determined by a comparative inspection of both parties. See also the same work, vol. iv. p. 365, 370.

As this is a crime of which the accusation is peculiarly easy, and the disproof proportionably difficult, more than ordinary acuteness is necessary for its investigation; and this can be best exercised while the event is recent, and before one or other of the parties can have time, deliberately, to frame the account of their injuries or innocence: here, as in some cases of murder, the medical practitioner is likely to be one of the earliest witnesses to the conduct of the accuser, (if not also of the accused,) immediately after the alleged transaction: to him, therefore, the Court will look, not only for surgical, but also for general observations. The following are among the first that will occur:

1st. What is the age, strength of body and mind, situation in life, and general character of the accuser?

2d. The same of the accused.

3d. Had the parties any, and what, previous acquaintance and intimacy?

4th. What external and obvious signs are there of violence?

5th. What surgical proof of coition, whether voluntary or violent?

6th. Is either party tainted by any, and what disease?

Time, place, and circumstances of the alleged offence.

A female may suffer violation at any age beyond absolute infancy; and the criminal records also furnish examples of brutality towards women of a very advanced period of life. As to the other sex, it may frequently be necessary to consider at what age a boy may be capable, or an old man incapable, of committing the offence. No determinate line can be drawn in either case; every instance must therefore rest upon its peculiar circumstances: this may, however, be allowed as a general rule, an attempt at violation is as extraordinary on the part of

---

young children, in consequence of a very general opinion among the lower libertines of the male sex, that the best possible cure for this disease is intercourse with a virgin: if, then, the accused should be found free from disease, where the female is contaminated, and *vice versa*, it affords a strong presumption of his innocence. In conducting, however, such an investigation, there are several sources of fallacy, with which it is the duty of the medical inquirer to be fully acquainted: he should know that purulent discharges, from other causes, do take place in children; and, on the other hand, that a person, in whom no appearance of existing venereal infection can be discovered, may communicate disease to others. This fact was ascertained by Mr. John Hunter, and its truth has been satisfactorily confirmed by the repeated observations of succeeding surgeons. Women labouring under leucorrhœa may impart a discharge to the male; and Dr. Male observes, that the latter, affected by a gleety discharge in consequence of strictures and other irritations in the urethra, may also affect the females.

extreme youth, as its completion is improbable in advanced old age. Sir M. Hale says, "A male infant under the age of fourteen is presumed by law incapable to commit a rape, and therefore, it seems, cannot be found guilty of it. For though in other felonies *malitia supplet ætatem;* yet, as to this particular species of felony, the law supposes an imbecility of body as well as of mind." This imbecility, however, is not universal, as we have previously shown when treating of the age of Puberty.

After having determined the age, the most material examination is as to the relative bodily strength of the parties. It is at all times difficult to believe that, in a mere conflict of strength, any woman of moderate power of body and mind could suffer violation, so long, at least, as she retained her self-possession. All accusation, therefore, must be viewed with suspicion, if there be not a great disparity of strength in favour of the assailant. But this remark must not be construed to extend to cases where, by long continued violence, intimidation, or other circumstances, the woman is ultimately overcome; for her mental sufferings may very considerably exhaust her power of resistance; "and it is no excuse or mitigation of the crime, that the woman at last yielded to the violence, and consented either after the fact or before, if such consent was forced by fear of death or duress." The mental power of the sufferer is also to be regarded: if it were considerable, greater power of resistance is to be expected; the contrary, if the woman were weak and timid; and, if she were actually imbecile, "a poor innocent that could not say him nay," the crime varies little or nothing in atrocity from the violation of an infant.

The external signs of violence ought to be inquired into upon the spot on which the crime is said to have taken place, and that as soon after the alleged commission as possible; that the state of surrounding objects may be determined, as well as the incidental injuries, as bruises, strains, &c. which either of the parties may have received in the struggle; the state of their clothes must be examined, and every circumstance, however minute, carefully noted. The case of Abraham Thornton, Warwick assizes, 1817, and the subsequent proceedings on the appeal in the King's Bench, Easter term, 1818, (1 *Bar. and Ald.* 405,) will show how material such examination may prove. Many of the observations to be made on cases of murder equally apply to those of rape; to them we must refer.

It is not necessary that the party violated should be proved a virgin up to the period of the alleged crime; for it may be committed on the person of a married woman, or of a widow; nay more, the law extends its protection against violence to those who have been notoriously unchaste: even a common strumpet is still under the protection of the law, and may not be forced; and it is not certain that she had not repented, and determined to reform. Yet, in the case of a person of notoriously bad reputation, the strongest possible evidence would be required to warrant a conviction.

"A very considerable doubt having arisen as to what shall be considered sufficient evidence of the actual commission of this offence, it is necessary to enter into an inquiry which would otherwise be offensive to decency. Considering the nature of the crime, that it is a brutal and violent attack upon the honour and chastity of the weaker sex, it seems more natural and consonant to those sentiments of laudable indignation which induced our ancient lawgivers to rank this offence among felonies, if all further inquiry were unnecessary after satisfactory proof of the violence having been perpetrated by the actual penetration of the unhappy sufferer's body. The quick sense of honour, the pride of virtue, which nature, to render the sex amiable, hath implanted in the female heart, as Mr. Justice Foster has expressed himself, is already violated past redemption, and the injurious consequences to society are in every respect complete. Upon what principle, or for what rational purpose, any further investigation came to be supposed necessary, the books which record the dicta to that effect do not furnish a trace."—(1 East's *P. C.* 436.)

But, on the other hand, it must be allowed that, as this is a crime peculiarly easy in accusation, and difficult in defence; and as experience has shown that prosecutions for this offence are very frequently resorted to from motives of revenge, malignity, disappointment, or extortion; the law has done well to extend its best protection to the possibly innocent, while it reserves its severest punishment for the truly guilty. It has occurred that there has not been the slightest ground for the accusation; that coition has never taken place, or been attempted by the party charged: the ordinary details are easily invented, and very colourable circumstantial evidence is soon obtained, by the designing accuser; it is only in the minuter points of examination, to which the present practice gives occasion, that she will trip in her evidence; it is to that only that the accused can look for safety when a well-forged tale, artfully compounded of truth and falsehood, is prepared for his destruction. Nor is it uncommon that a woman, who has actually consented to her own dishonour, should, on fear of discovery, or on disappointment, or from jealousy, prefer an accusation of rape against her seducer: here the main fact being true, the coition having taken place, and under the usual circumstances of secresy, the life of a prisoner depends on the mere question of consent or violence. The prosecutrix being the principal, or more generally the only, witness, it is essential that her testimony should be subjected to the most rigid examination, and that all external circumstances should be sought which might tend to confirm or destroy it.

The first and most material point to be proved is, that the venereal congress or coition has actually taken place; but, as to the exact legal definition of this act, much difference of opinion has existed; for, while some learned authorities have held that penetration alone is necessary, others have maintained that the crime is not perfected without *emissio seminis* also. Lord Coke, defining "carnal knowledge," says there must be *penetratio*, that is, *res in re;* but the least penetration maketh it carnal knowledge.* So, in the case of Russen the schoolmaster, it

---

* Many of the judges denied that carnal knowledge was necessary to be laid in the indictment; but only that the defendant ravished the party.—(*Hill's* case, Tr. Term, 1781.)

was proved by two surgeons, on behalf of the prisoner, and corroborated by four others who had examined the girl, that the hymen (which they considered an indubitable mark of virginity,)* was whole and unbroken, and that the passage was so narrow that a finger could not be introduced. But it was admitted that this membrane, the existence or non-existence of which has been strongly controverted,† was, in some

---

* M. Capuron in his "Medicine Legale relative a l'Art des Accouchemens," published at Paris, 1821, enters with some minuteness into the discussion of these signs; and comes to the conclusion, that we shall endeavour to impress upon the reader, that no one of the signs is in itself sufficient to establish the fact; nor is the absence of all conclusive against its existence: all that the most experienced medical observer can do, is to show a strong probability, which, united to moral evidence of the character and conduct of the party, will amount to proof.—Χρη παντα θεασασθαι τα σημεια, και μη πιστευειν ενι.

† In entering upon a disquisition on the tests of virginity, it is hardly necessary to enumerate the many absurd marks related by the more credulous, as indicative of recent defloration,—such as swelling of the neck, rings around the eyes, the colour of the skin and urine, &c.; nor is it necessary to enter into a refutation of the story, credited by Mahon, of a monk at Prague who could tell a maid by the smell. We shall therefore proceed at once to consider the value of that test which most commonly passes among us as the least equivocal mark of virginity,—viz. the presence of a peculiar membrane termed the *hymen*.

The Hymen (so named from the Greek word ὑμην, a membrane,) is formed by four angular duplicatures of the membrane of the vagina, the union of which may be discovered by corresponding lines on the hymen. At the upper part there is a semilunar vacancy, intended for the transmission of the menses, so that it assumes the form of a crescent; a circumstance which affords the true explanation of the origin and meaning of the symbol so characteristically assigned to Diana. In some rare cases, the hymen is an imperforate circular membrane, attached to the edge of the orifice of the vagina in every part, so as to close the canal completely. The girls, in whom this fault of conformation existed, were called by the Greeks ἀτρηται; the physicians who have written in Latin amongst us have given them the name of *Imperforatæ*, *clausæ*, or *velatæ*; and the Italians that of *Coperchiate*. The Romans had no appropriate word to denote this malformation, and they were therefore obliged to express it by some circumlocution: it is thus that Cicero (*De Divinat.* lib. ii.) speaks of a dream, where a woman was seen "*quæ obsignatam habebat naturam*;" and that Pliny (*Hist. Nat.* lib. vii. c. 16,) relates, Cornelia, the mother of the Gracchi, "*concreta genitali nata fuerat*." In many cases the membrane appears never to have been formed; while, in others, its extreme tenacity has occasioned its rupture and destruction in early life: it may, moreover, have been destroyed by disease, by noxious habits, or by acrimonious discharges. This extreme uncertainty has led many authors, of no inconsiderable eminence, to deny its existence; while others have acknowledged its occasional presence, but have attributed its formation to disease. Graaf, Penius, Buffon, Dionis, declare that, by dissection of girls of all ages, they have never been able to discover it: on the other hand, the reality of this membrane has been maintained by Berenger de Carpi (*In Isagoge Anatomica*), Vesalius (*De Corp. Hum. Fabric.* v. c. 15), Fallopius (*In Observat. Anatom.*) Volcherus Coiterus (*In Tabul. Anatom.*), Varolius (*Anatom.* lib. iv. c. 4), Riolanus (*Authropog.* lib. i. c. 16), Bartholin (*Anat.* lib. i. c. 31), Weirus (*Observat.* lib. i. *et de Lamiis*, lib. iii. c. 20), Spigelius (*De Hum. Corp. Fabrica*, lib. viii. c. 18), Diemerbroeck (*Anatom.* lib. i. c. 16), Swammerdam (*De Uteri Mulieb. Fabrici*), Techmeyer (*Institut. Medicin. Legal et Forens.* c. iv.), and all the more learned and able anatomists of the sixteenth and seventeenth centuries. Heister (*Compend. Anatom.* and *Ephem. Nat. Curios. Cent.* viii. *Observ.* 69), Frederic Ruysch (*Thes. Anatom.* iii. No. 15; vi. No. 1; vii. No. 60), Morgagni (*Adversaria Anatom.* i. 29; iv. 23), and Winslow (*Exposit. Anatom.* No. 653), all describe this membrane, and assert that they have found it in every young girl they have had occasion to examine. Astruc (*on the Diseases of Women*, vol. i. p. 123), in referring to the above learned instances situated an inch or an inch and a half beyond the vagina;* and Mr. Justice Ashhurst, who tried the prisoner, left it to the jury whether any penetration were proved; for, if there were any, however

---

authorities, observes that "the inference must necessarily be, that those who deny ever to have seen it, must either have examined only such girls as had lost their virginity, or, prepossessed with the false notion that the hymen must always close the entrance to the vagina entirely, they have mistaken it at the time it was before their eyes, and have even sometimes given the description of it, without mentioning the name." After this literary history of the question, we may very safely conclude that the hymen is a perfectly natural structure, occurring in the virgin, and that by sexual intercourse it is ruptured; after which it is shrivelled into several small excrescences at the orifice of the urethra, called the *carunculæ myrtiformes*. But, since it is liable to such variations in appearance, and to accidental rupture from the slightest causes, its absence can never be received as evidence of defloration; nor can its presence be considered as an unequivocal proof of virginity; for it has been asserted by indisputable authority, that it is not always ruptured *in coitû*. Ruysch has said, that, if the coitus take place immediately after the menstrual excretion, this membrane is often not ruptured, (*Observ. Anat. Chirurg.* xxii.) And we have already alluded to cases wherein the hymen was actually entire at the time of delivery.

Some authors have talked of the renewal of the hymen after its rupture: this, we apprehend, can never happen, although a spurious reparation of certain local consequences, incident to the loss of virginity, may certainly occur from the effects of adhesive inflammation.

Having thus disposed of the subject of hymen, we next come to consider the state of the Vagina as an indication of virginity, upon which some authors have attached considerable weight, especially the Italian medico-jurist Tortosa. In a healthy virgin it ought certainly to be rigid and narrow, since the only function which it has to perform is that of giving transit to the menstrual flux: the parts may, however, become dilated, and their natural rugæ be obliterated, from various innocent causes. Certain mal-practices will likewise occasion the same relaxation as sexual intercourse. The Mosaic test of virginity, the effusion of blood, however conclusive it might have been among the Jews, certainly cannot be received as unexceptionable in these northern climates. The Jews, it would seem, placed so much reliance upon appearances, that the nuptial sheets were constantly viewed by the relations on both sides; and the maid's parents preserved them as a token of her virginity, to be produced in case her husband should ever reproach her upon that subject. In case the token of virginity was not found on them, she was to be stoned to death at her father's door. This evidence is still required by some of the tribes inhabiting the banks of the Indus. (Pottinger's *Travels*, p. 70.) In some cases the effusion of blood during the first act of coition is very considerable, and is liable to be confounded with the catamenia: we have, however, already observed that the menstrual excretion does not, in its natural state, coagulate; and yet this assertion requires some qualification, for it is well known that, when the discharge is superabundant and attended with great pain, it often comes away in coagula, in which case there is probably an admixture of common blood.

From what has been here related, we are bound to conclude that there does not exist any anatomical sign by which the virginity of a female can be unequivocally determined. By midwives and matrons, however, the subject has been treated with less diffidence: in the statutes of the sworn matrons, or midwives of Paris, containing likewise divers formulæ of reports, and depositions made in court, upon their being called to visit girls that made their complaint of being deflowered, they laid down fourteen marks on which to form a judgment. Laur. Joubart, a celebrated physician at Montpellier, has transcribed three of these reports; one made to the provost of Paris, another in Languedoc, and a third in Berne.

---

* Mahon mentions an instance in which he found a membrane at a finger's breadth within the vagina. (*Med. Leg.* tom. i. p. 118.)

small, the rape was complete in law. The jury found him guilty, and he received judgment of death. But, before the time of execution, the matter being much discussed, the learned judge reported the case to the other judges for their opinions, whether his direction were proper? and, upon a conference, it was unanimously agreed by all assembled, (in the absence of C. J. De Grey and B. Eyre,) that the direction of the judge were perfectly right. They held that, in such cases, the least degree of penetration is sufficient, though it may not be attended with the deprivation of the marks of virginity: it was therefore properly left to the jury by the judge; and accordingly the prisoner was executed. This decision appears to be well warranted by physiological observation; for, as it is evident, from the concurrent testimony of the highest medical authorities, that penetration in vaginam is not necessary to conception, it would be absurd to contend that more were necessary to constitute rape in law than generation in nature. The utmost wrong to the one party, and the malignant intent of the other, have been complete; and the injury on the one hand, and malice on the other, are truer criteria for the administration of justice, than the dicta of lawyers or the etymologies of schoolmen.

Lord Coke, (12 *Rep.* 37,) Sir M. Hale in his Summary, and Hawkins (*P. C.*), say that there must be both *penetratio* and *emissio seminis;* and this appears to be the law of the present day, as decided by Skynner, C. B. Gould, Willis, Ashhurst, Nares, Eyre, and Hotham, against Lord Loughborough, Buller, and Heath; Lord Mansfield, though present, having given no opinion of his own, (a circumstance from which we might infer that he agreed with the minority.) The argument is stated to have turned on the words *carnal knowledge*, to which the majority contended that *emissio seminis* was absolutely necessary: if, therefore, it be true that certain eunuchs have the power of erection, and consequently of penetration, they may morally ravish without incurring the punishment of rape, for it is certain that they can have no *emissio seminis;** or a man may have perpetrated all the more atrocious parts of his crime, and yet, being interrupted in the least voluntary constituent of it, (Hill's case,) escape the well-merited vengeance of the law; while it is evident, on the other hand, that the innocent victim has suffered, in body, mind, and reputation, as much as if the crime had been legally completed.

But, admitting the fact of emission to be necessary to the constitution of this crime, it remains to inquire whether the proof of this fact must be specifically made out in evidence, or whether it shall be presumed. In Matthew Cave's case, (Oct. 1747,) Chief Justice Willes directed the prisoner to be acquitted for want of proof; but, on the other hand, Mr.

* An important question here arises as to what shall be legally called semen; for the secretion emitted is composed of parts, the smaller portion of which only possesses the generative faculty.

It appears, from the experiments and observations of our most accurate physiologists, that the fluid expelled in copulation is furnished in a small proportion only by the testes; that to this a peculiar secretion of the vesiculæ seminales is added, and that the chief bulk is made up of the prostatic liquor, or secretion from the prostate gland; so that the fact of emission in eunuchs is not extraordinary, although the discharged fluid cannot be said to be seminal.

Justice Foster, J. Clive, (in Blomfield's case, A.D. 1758,) J. Bathurst, and Baron Smythe, (in Sheridan's case, 8 Geo. III.) and J. Buller, (in Harmwood's case, Winchester Spring assizes, A.D. 1787,) held the contrary. The latter case is the more worthy of consideration, as it was subsequent to the decision in Hill's case, and tried by one of the judges present at the discussion. "He said, in giving judgment, that he recollected a case where a man had been indicted for a rape, and the woman had sworn that she did not perceive any thing come from him; but she had had many children, and was never in her life sensible of emission from a man:* and that was ruled not to invalidate the evidence which she gave of a rape having been committed upon her." (1 East's *P. C.* 440.)

A rape may have been committed on a child too young, or rather too incompetent, to be sworn; yet all the circumstances, except this, may be proved by other witnesses: the infant alone could prove *emissio in vaginam*, for no subsequent examination, however immediate, would demonstrate the fact; or when a woman has fainted from the violence committed on her, or has been dishonoured in her sleep,† and through the agency of soporific drugs, or has died before the trial,‡ or been murdered by her ravisher, or has been driven to suicide by mental distraction; in all these cases of increased atrocity this mode of proof becomes impossible.

But emission, it is said, may be presumed from penetration, (Duffin's case, June 1821;) but this is not physiologically true in all cases, and as we have stated that it may be prevented by accident or interruption, so also emission is said to be evidence of penetration; but this is still less reasonable, for it is obvious that it may easily occur in the mere attempt: yet, if reliance can be placed on the authorities already quoted,§ emission alone, without any material penetration, but only by injection *inter labia*, will be sufficient to impregnate, and therefore ought in reason to be considered sufficient to constitute the crime of rape.

When it has been clearly proved that coition has actually taken place between the parties charged, the next point to be determined is, whether the woman consented or not. It is not necessary that we should here enter into a detail of all the circumstances which may throw light on this question; but one extraordinary dictum of the more ancient lawyers is worthy of observation, though there is little fear that the error will ever be sanctioned by any tribunal; yet, as it is one of the

---

* We should, indeed, be inclined to question the veracity of a witness, who, under circumstances of extreme pain, rage, and terror, should pretend to any very great sensibility to minuter accidents.

† The Faculty of Leipsic decided, "Dormientem in sella virginem insciam deflorari posse," (1 Valent. Pand. *Med. Leg.* p. 31, *vide etiam ib.* p. 33.) "De stupris in somno à feminis admissis." In stating the above authorities, we are not to be considered as implicitly confiding in their truth.

‡ Yet, if she live long enough to make a deposition upon oath, it is admissible.

§ In the celebrated case of Mary Ashford, the prisoner, Abraham Thornton, admitted the carnal knowledge, adding that it was with her own consent; but the whole of the evidence repelled the latter assertion: the death of his unhappy victim, however caused, rendered it impossible to convict him of rape.

evils of this crime that an unmerited stigma too frequently attaches to the sufferer by it, we are the more anxious to expose the vulgar idea, from which some ignorant persons might still infer that a woman had consented, because she had proved pregnant. "It is said by Mr. Dalton, that if a woman, at the time of the supposed rape, do conceive with child by the ravisher, this is no rape; for, he says, a woman cannot conceive unless she doth consent. And this he hath from Stamford and Britton, and Finch. Dalt. c. 160; see also 2 Inst. 190.* But Mr. Hawkins (*P. C.* c. 41, s. 2,) observes that this opinion seems very questionable; not only because the previous violence is in no way extenuated by such a subsequent consent, but also because, if it were necessary to show that the woman did not conceive,† the offender could not be tried till such time as it might appear whether she did or not; and, likewise, because the philosophy of this notion may be very well doubted of. (1 Hawk. 108.) And Lord Hale says, this opinion in Dalton seems to be no law. (1 H. H. 131;) see also *Mss. Sum.* 334.) That so absurd a notion as that conception evidenced consent, should in modern times have obtained amongst any whose education and intellect were superior to those of an old nurse, is indeed surprising: at this day, however, facts and theory concur to prove that the assentation of nature in this respect is no ways connected with violation of mind." (Burn's *Just.* tit. *Rape.*)

It is not necessary that the quantum of violence be extreme; it is sufficient that the offence is committed without consent; as where a woman is violated in her sleep or during a fit, and query if she have been intoxicated for that special purpose, so that in truth she should have no rational power to consent or deny; or if the ravisher imposed himself in the night on a married woman as her husband.

If a woman be compelled by violence to marry, and carnal knowledge be had by force, it is a rape, (1 Hale, 629;) but, as there is another remedy, by statute 3 Hen. VII. c. 2, for the forcible abduction, it is not necessary to inquire whether an indictment will lie, until the marriage be dissolved.

Nor will a subsequent marriage purge the offence: formerly "it was held for law that the woman, by consent of the judge and her parents, might redeem the offender from the execution of his sentence, by accepting him for her husband, if he also was willing to agree to the exchange, but not otherwise." (Glanv. l. 14, c. 6; Bract. l. 3, c. 28;) and this was reasonable while the prosecution was at the suit of the party by appeal; for, as the king could not pardon, the power of remission might be properly left to the person injured; but, that outrages might not be too readily compromised to the injury of public justice, the statute 6 Rich. II. st. 1, c. 6, enacts, that the woman consenting,

---

* Farr and Faselius incline to the same opinion. The Parliament of Thoulouse passed a decree upon this subject, deciding that a woman violated might nevertheless conceive; the physicians having on that occasion reported, "Posse quidem voluntatem cogi, sed non naturam, quæ semel irritata pensi voluptate fervescit, rationis et voluntatis sensum amittens."

† Or, if she be a married woman, how is it possible to fix the filiation?

and the ravisher, be "disabled to challenge all inheritance, dower, or joint feoffment, after the death of their husbands and ancestors," and the husband, or, if she have none, the father or next of blood, shall have the appeal. But, rape having been made felony by statute West. 2, c. 34, and a new appeal given, the option of the woman is now taken away. It would have been unnecessary to have dwelt on this point, if a vulgar error did not to this day prevail among the lower orders, that the punishment of rape might be escaped by the connivance of the nominal prosecutrix, even after judgment.

"The party grieved is so much considered as a witness of necessity in this, as in other personal injuries, that, in Lord Castlehaven's case, who assisted* another man in ravishing his own wife, she was admitted as a witness against him.

And, if the party be dead, "the deposition of the girl taken before the committing magistrate, and signed by him, may, after her death, be read in evidence at the trial of the prisoner, although it was not signed by her, and she was under twelve years of age; provided she was sworn, and appeared competent to take an oath, and all the facts necessary to complete the crime may be collected from the testimony so given in evidence." (The King against Fleming and Windham, A. D. 1779; Leach's *C. L.* p. 996.) But, if the declaration be made in *articulo mortis*, the party knowing herself to be dying, then it is not necessary that she be sworn, for the solemnity of the occasion is more than equivalent to the form of an oath; yet it is necessary that the party should have so much sense and discretion that, if in sound health, she might have been sworn; for, if she have not, then even the fear of death and judgment may not have a sufficient impression on her mind. The melancholy case of Coleman will impress every reader with the importance of carefully noticing the circumstances of dying declarations, lest, by receiving as evidence the ravings of delirium, or at least the imperfect impression of impaired faculties, the innocent should be sacrificed to the errors of the dying; and this is the more necessary in those cases where the atrocity of the crime committed creates an immediate prejudice against every party charged or suspected.

---

* All persons, whether men or women, aiding in the perpetration of a rape, are guilty of felony. (Lord Baltimore's case, 2 Burr. 2179.)

